# Effects of physical and psychological symptoms on cancer-related fatigue among esophageal cancer patients

**DOI:** 10.1186/s12885-024-12138-4

**Published:** 2024-03-29

**Authors:** ChunYing Cui, Lie Wang, XiaoXi Wang

**Affiliations:** 1 School of Humanities and Management, Wannan Medical College, 241002 Wuhu, Anhui PR China; 2 School of Public Health, China Medical University, No.77 Puhe Road, Shenyang North New Area, 110122 Shenyang, Liaoning PR China; 3 Medical Basic Experimental Teaching Center, China Medical University, No.77 Puhe Road, Shenyang North New Area, 110122 Shenyang, Liaoning PR China

**Keywords:** Esophageal cancer, Physical symptoms, Depressive symptoms, Anxiety, Cancer-related fatigue

## Abstract

**Background:**

Cancer-related fatigue (CRF) is considered one of the most prevalent and distressing symptoms among cancer patients and may vary among patients with different cancer types. However, few studies have explored the influence of physical and psychological symptoms on CRF among esophageal cancer (EC) patients without esophagectomy. Therefore, this study aimed to examine the effects of physical and psychological symptoms on CRF among EC patients without esophagectomy.

**Methods:**

In the present study, a cross-sectional study was conducted from February 2021 to March 2022 in Liaoning Province, China. Among the 112 included participants, 97 completed our investigation. The questionnaires used consisted of the Brief Fatigue Inventory (BFI), the MD Anderson Symptom Inventory Gastrointestinal Cancer Module (MDASI-GI), the Patient Health Questionnaire-9 (PHQ-9), the Generalized Anxiety Disorder-7 (GAD-7), and demographic and clinical information. Multivariate linear regression was conducted to test the relationships between physical and psychological symptoms and CRF.

**Results:**

Of the 97 EC patients, 60.8% reported CRF (BFI ≥ 4). The mean age of the participants was 64.92 years (SD = 8.67). According to the regression model, all the variables explained 74.5% of the variance in CRF. Regression analysis indicated that physical symptoms, including constipation, diarrhoea, and difficulty swallowing, contributed to CRF. On the other hand, depressive symptoms increased the level of CRF among EC patients without esophagectomy.

**Conclusions:**

Given the high prevalence of CRF among EC patients without esophagectomy, it is urgent to emphasize the importance of fatigue management interventions based on physical and psychological symptoms to alleviate CRF in EC patients.

## Introduction

According to CLOBOCAN 2020 [1], EC is the seventh most commonly diagnosed cancer and the sixth leading cause of cancer death. Eastern Asia has the highest regional incidence rates for both men and women, mainly due to China’s enormous burden [2]. EC is characterized by an increasing incidence, demanding treatment, and a poor prognosis [3]. In diagnosis and therapy processes, EC patients would experience the cancer- and treatment-related side effects that could have a negative influence on overall quality of life [4]. Cancer-related fatigue (CRF) is considered one of the most prevalent and distressing symptoms among cancer patients [5–7].

Cancer-related fatigue (CRF) is characterized mainly by a distressing, persistent, subjective sense of physical, emotional, or cognitive tiredness or exhaustion related to cancer or cancer treatment; this condition cannot be alleviated by rest or sleep and interferes with the usual functioning [8–10]. CRF significantly affects EC patients’ survival and prognosis. Previous study reported that approximately 60% of EC patients experience CRF [11]. In addition, studies demonstrated that the severity of CRF among EC patients with esophagectomy was significantly higher than those without surgery [4]. Postoperative complications were associated with increased CRF after esophagectomy [12]. However, few studies assess the level of CRF and explore its influencing factors among EC patients without esophagectomy.

Although the exact mechanism of CRF has not been identified within the cancer context, an increasing number of studies have indicated that the causes of CRF are multifactorial and include physical, psychological, sociodemographic, and cultural factors [13]. Physical factors include pain, altered energy metabolism, and anemia-related neuroendocrine changes. Fatigue is associated with a high symptom burden and higher severity of physical symptoms [14]. On average, cancer patients reported 8 to 13.5 symptoms, depending on the cancer type and care level [15, 16]. EC patients are susceptible to persistent and moderate-to-severe gastrointestinal symptoms related to cancer, particularly difficulty swallowing. Therefore, the physical symptoms of the patients included in this study were gastrointestinal cancer-related symptoms, including constipation, diarrhoea, difficulty swallowing, change in taste, and abdominal distention.

Depression and anxiety are the most common psychological problems among cancer patients. A previous study revealed that the prevalence of anxiety and depression was 34% and 23%, respectively, among EC patients before surgery [17]. Previous research has shown that depression and anxiety are significantly associated with fatigue in patients with other cancer types [18]. Ohkura et al. also reported that psychological distress (e.g., depression and anxiety) significantly influenced health-related quality of life (HRQOL) among EC patients [19]. In addition, a prospective cohort study indicated that more severe depressive symptoms were associated with more activity limitations and dysphagia symptoms severity in EC patients [20]. Therefore, in the present study, the psychological symptoms included depressive and anxiety symptoms.

Although more studies have explored the effect of CRF on psychological distress, reciprocal relationships (fatigue → psychological distress → fatigue) have been ignored. Additionally, few studies explore the influencing factors of CRF among EC patients without esophagectomy. Therefore, our study aimed to investigate the impacts of physical and psychological symptoms on CRF in EC patients without esophagectomy.

## Methods

### Design and participants

The cross-sectional study was conducted from February 2021 to March 2022 in Liaoning Province, China. Using a nonrandom sampling method, participants were recruited from the Department of Radiation Oncology, the First Affiliated Hospital of China Medical University. The inclusion criteria were (1) patients diagnosed with EC, (2) patients received no esophagectomy treatment, (3) patients with knowledge of their disease, (4) patients aged at least 18 years or older, and (5) patients who were able to communicate and write in Chinese. Patients with a history of mental or cognitive disorders and other diseases, such as gastrointestinal diseases and other cancers, were excluded. Eligible participants were included in the present study. In total, 112 patients met our inclusion criteria and provided written informed consent to participate in our research. Ultimately, 97 valid questionnaires were recovered after excluding 15 participants with poor answer responses. The procedures used in our survey were approved by the Committee on Human Experimentation of the First Affiliated Hospital of China Medical University (NO. 2021-430-2).

### Measures

#### Cancer-related fatigue

The Chinese version of the Brief Fatigue Inventory (BFI) [21] was used to measure CRF in patients with EC, a reliable instrument for rapidly assessing fatigue levels among cancer patients. The scale contains nine items. Specifically, three items ask patients to evaluate fatigue severity “right now,” “usual” fatigue in the past 24 h, and the “worst” fatigue in the past 24 h, with 0 indicating “no fatigue” and 10 indicating “fatigue as bad as you can imagine.” Six items are used to rate the amount of interference with function (different aspects of life) caused by fatigue in the past 24 h. The items regarding interference comprise general activity, mood, walking ability, regular work, relationships with other people, and enjoyment of life, and each item is scored on a score from 0 to 10, ranging from “does not interfere” (0) to “completely interferes” (10). A global score is calculated by taking the average of the nine items [22], with a cut-off point ≥ 4 indicating moderate to severe fatigue [21]. The internal consistency (Cronbach’s alpha coefficient) in the present study was 0.974.

#### Physical symptoms

The MD Anderson Symptom Inventory Gastrointestinal Cancer Module (MDASI-GI) [23] was used to assess gastrointestinal cancer-specific symptoms. The scale includes a 13-item core symptoms subscale and a five-item gastrointestinal cancer-specific subscale. In the present study, the five-item subscale was used to measure EC patients’ physical symptoms, including constipation, diarrhoea, difficulty swallowing, change in taste, and abdominal distention, and each symptom was scored on an 11- point Likert type (0= “not at all” to 10= “as bad as you can imagine”). A higher score indicates more severe physical symptoms among EC patients. Chen et al. [24] translated the scale into Chinese. The Cronbach’s α coefficient for the specific symptom subscales was 0.866 in this study.

#### Psychological symptoms

In the present study, the psychological symptoms included depressive and anxiety symptoms.

Depressive symptoms were assessed using the Chinese version of the Patient Health Questionnaire-9 (PHQ-9) [25]. The PHQ-9 includes nine items rated on a four-point scale ranging from 0 (never) to 3 (almost every day). A higher score indicates a greater frequency of depressive symptoms, with a cut-off point ≥ 7 indicating depression. The Chinese version of the PHQ-9 has been found to have good reliability and validity among Chinese cancer patients [26]. The alpha coefficient for the PHQ-9 was 0.873 in the present study.

Anxiety symptoms were assessed using the Chinese version of the Generalized Anxiety Disorder-7 (GAD-7) [27]. The GAD-7 comprises seven items rated on a four-point scale ranging from 0 (never) to 3 (nearly every day). A higher score indicates more severe anxiety, with a cut-off point ≥ 5 indicating anxiety. The Chinese version of the GAD-7 has been used widely among Chinese cancer patients [28]. The alpha coefficient for the GAD-7 was 0.926 in the present study.

#### Demographic and clinical information

Demographic data, including age, sex, educational background, marital status, employment status, family per capita monthly income (CNY), smoking status, alcohol consumption status, and physical exercise (PE) level, were collected using a questionnaire. Clinical data, including lymph node metastasis status, time since diagnosis, and family history, were obtained from medical records.

### Statistical analysis

Independent t- tests and one-way ANOVAs were used to examine the relationships among demographic information, clinical characteristics, and CRF. The correlation coefficient (*r*) of continuous variables (CRF, and physical and psychological symptoms) was tested using Pearson’s correlation analysis. Hierarchical linear regression was conducted to explore the influence of the control variables and physical and psychological symptoms on CRF at each step. The control variables were entered in Step (1) Our study included age, physical exercise (PE) level, and time since diagnosis in the model as potential confounders. Physical symptoms, including constipation, diarrhoea, difficulty swallowing, change in taste, and abdominal distention, were added as independent variables in Step (2) In Step 3, psychological symptoms (depressive and anxiety symptoms) were included.

SPSS version 20.0 was used for statistical analysis, and *P* < 0.05 was considered to indicate statistical significance.

## Results

### Characteristics of participants

The mean age of all participants was 64.92 (SD = 8.67) years, and 60- to 70-year-old patients tended to have a greater level of CRF than did those younger than 60 years and older than 70 years (*F* = 3.740, *P* = 0.027). More than 94.0% of the patients were males, and 86.6% were married/cohabiting. Approximately 34.0% of patients had a senior high school education or above, and 22.7% had a primary school education or lower. Among the patients, 64.9% were retired, and 9.3% were full-time employees. Approximately 77.0% of patients had a family per capita monthly income lower than 3000 (CNY). A total of 79.4% and 84.5% of the patients had a history of smoking and alcohol consumption, respectively. More than 81% of patients performed PE (≥ 1 times/week), and patients who did not perform PE reported higher CRF scores (*F* = 4.109, *P* = 0.019) than did those who performed PE. More than 71% of patients had no family history of cancer and 29.9% had lymph node metastasis. Overall, 69.1% of the patients were diagnosed less than one year ago and had lower CRF scores (*F* = 3.362, *P* = 0.039) than did those diagnosed more than one year ago. Therefore, in our study, age, PE level, and time since diagnosis were chosen as control variables for the hierarchical multiple linear regression analysis. The detailed results are presented in Table 1.

**Table 1:** Demographic and clinical characteristics associated with cancer-related fatigue (CRF) among EC patients without esophagectomy

Variables	N (%)	CRF (Mean±SD)	t/F	*P*
Gender			0.295	0.769
Male	92 (94.8)	3.26±2.82		
Female	5 (5.2)	3.64±3.62		
Age			3.740	0.027
<60	30 (30.9)	2.72±2.28^b^		
60–70	40 (41.2)	4.19±3.18^a^		
≥70	27 (27.8)	2.54±2.61^b^		
Marital status			0.260	0.796
Married/cohabited	84 (86.6)	3.31±2.85		
Single or others	13 (13.4)	3.09±2.97		
Educational level			0.596	0.553
Primary school or under	22 (22.7)	3.85±2.98		
Middle school	42 (43.3)	3.16±2.73		
Senior high school or above	33 (34.0)	3.04±2.94		
Employment status			0.725	0.487
Full-time	9 (9.3)	2.60±2.42		
Part-time/unemployment	25 (25.8)	3.80±3.06		
Retire and others	63 (64.9)	3.16±2.82		
Monthly household income			0.459	0.634
<2, 000	35 (36.1)	3.38±2.79		
2, 000–3, 000	40 (41.2)	3.47±2.88		
>3,000	22 (22.7)	2.77±2.94		
Smoking			0.732	0.466
No	20 (20.6)	2.86±2.76		
Yes	77 (79.4)	3.39±2.88		
Alcohol consumption			0.759	0.450
No	15 (15.5)	2.76±2.93		
Yes	82 (84.5)	3.37±2.84		
Physical exercise (week)			4.109	0.019
0	18 (18.6)	4.87±3.14^a^		
1–2	17 (17.5)	2.37±2.73^b^		
≥ 3	62 (63.9)	3.06±2.65^b^		
Lymph node metastasis			0.661	0.729
No	55 (56.7)	3.95±2.95		
Yes	29 (29.9)	2.69±2.66		
Missing	13 (13.4)	2.71±2.59		
Time since diagnosis			3.362	0.039
<1 year	67 (69.1)	2.79±2.42^a^		
1–2 years	17 (17.5)	4.19±3.42^b^		
≥2 years	13 (13.4)	4.58±3.56^b^		
Family history			0.192	0.826
No	69 (71.1)	3.16±2.81		
Yes	11 (11.3)	3.60±3.07		
Unknown	17 (17.5)	3.54±3.02		

a, b: Calculated by least-significant-difference (LSD), mean scores for CRF with unequal superscripts differ significantly at the *p* < 0.05 level

CRF, cancer-related fatigue; SD, standard deviation

### Correlations among continuous variables

Table 2 presents the correlations between CRF and physical and psychological symptoms. Physical symptoms, including constipation, diarrhoea, difficulty swallowing, change in taste, and abdominal distention, were positively correlated with CRF (*r* = 0.583–0.693, *P* < 0.01). Additionally, psychological symptoms, including anxiety and depressive symptoms, were positively related to CRF (*r* = 0.627–0.657, *P* < 0.01).

**Table 2:** Descriptive statistics and correlations analysis

Variables	CO	DI	DS	CT	AD	DE	AN	CRF
Constipation (CO)	1							
Diarrhea (DI)	0.647^**^	1						
Difficulty swallowing (DS)	0.500^**^	0.445^**^	1					
Change in taste (CT)	0.533^**^	0.603^**^	0.617^**^	1				
Abdominal distention (AD)	0.613^**^	0.556^**^	0.554^**^	0.687^**^	1			
Depression (DE)	0.383^**^	0.220^*^	0.460^**^	0.400^**^	0.467^**^	1		
Anxiety (AN)	0.410^**^	0.198	0.414^**^	0.237^*^	0.420^**^	0.790^**^	1	
Cancer-related fatigue (CRF)	0.679^**^	0.583^**^	0.693^**^	0.604^**^	0.646^**^	0.657^**^	0.627^**^	1
Mean	3.02	1.93	5.25	2.77	2.51	5.47	3.92	3.28
SD	3.11	2.47	3.70	3.12	3.08	5.83	5.03	2.85

**, *P* < 0.01; *, *P* < 0.05

SD, standard deviation

### Results of the hierarchical multiple linear regression

**Fig. 1:**
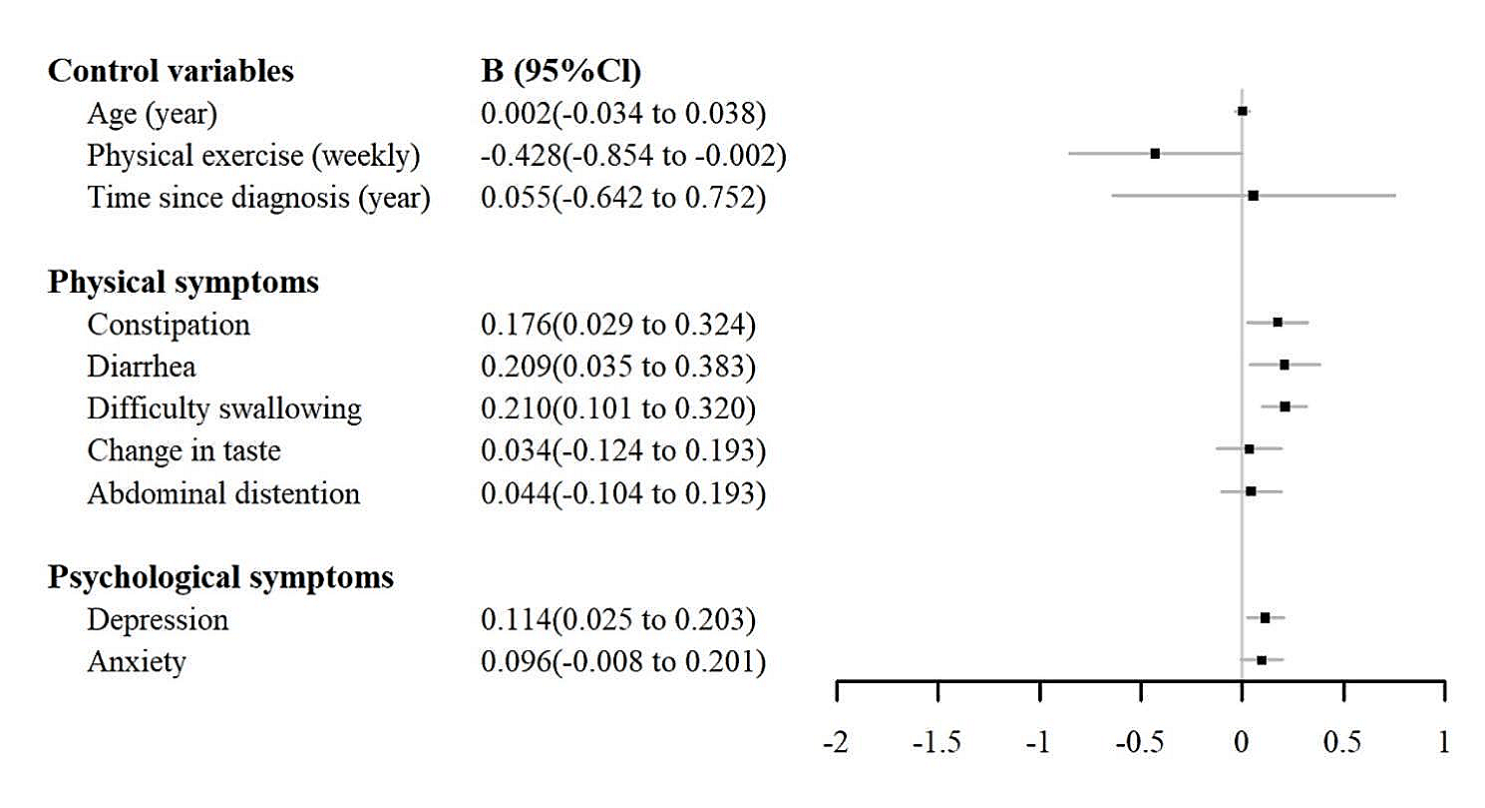
Forest plot of the associated factors of cancer-related fatigue (CRF) among EC patients without esophagectomy. (Hierarchical multiple linear regression)

Table 3 indicates that the regression model explained 74.2% of the variance in CRF. The *R*^2^ changes demonstrated that the incremental variances explained by each block were 10.7%, 57.1%, and 9.4% for demographic and clinical characteristics, physical symptoms, and psychological symptoms, respectively. According to the final linear regression model and forest plot (Fig. 1), PE (*β*=-0.108, *P* = 0.045) decreased the risk of CRF. In addition, constipation (*β* = 0.192, *P* = 0.020), diarrhoea (*β* = 0.181, *P* = 0.019), difficulty swallowing (*β* = 0.273, *P* < 0.001), and depressive symptoms (*β* = 0.233, *P* = 0.013) increased the risk of CRF among EC patients without esophagectomy.

**Table 3:** Hierarchical multiple linear regression analysis of cancer-related fatigue among EC patients without esophagectomy

Variables	Cancer-related fatigue (CRF)						
	B	95% CI	t	*P*	β	Adjusted R^2^	ΔR^2^
**Demographic and clinical characteristics**						0.068	0.107
Age	0.002	-0.034 to 0.038	0.094	0.925	0.005		
Physical exercise (week)	-0.428	-0.854 to -0.002	2.010	0.045	-0.108		
Time since diagnosis (year)	0.055	-0.642 to 0.752	0.157	0.875	0.029		
**Physical symptoms**						0.644	0.571
Constipation	0.176	0.029 to 0.324	2.372	0.020	0.192		
Diarrhea	0.209	0.035 to 0.383	2.391	0.019	0.181		
Difficulty swallowing	0.210	0.101 to 0.320	3.818	< 0.001	0.273		
Change in taste	0.034	-0.124 to 0.193	0.430	0.668	0.038		
Abdominal distention	0.044	-0.104 to 0.193	0.593	0.555	0.048		
**Psychological symptoms**						0.742	0.094
Depression	0.114	0.025 to 0.203	2.539	0.013	0.233		
Anxiety	0.096	-0.008 to 0.201	1.832	0.070	0.170		

B, unstandardized beta; β, standardized regression weight; CI, confidence interval

## Discussion

CRF is a very disabling and distressing symptom that affects EC patients. The present study investigated the prevalence of CRF and examined the associations of cancer-related physical and psychological symptoms with CRF among EC patients without esophagectomy. Our study revealed that the prevalence of CRF was 60.8% (cut-off ≥ 4) among Chinese EC patients based on the BFI score, which was greater than that reported in previous research conducted among patients with other cancer types. For example, Ouyang et al. [29] reported that the prevalence of CRF was 51.4% in colorectal cancer patients using the BFI score (≥ 4 points). Another study by Hwang et al. [30] reported that 51.3% of stomach cancer survivors had moderate-severe CRF based on the BFI score. In addition, Hung et al. [31] reported that the prevalence of CRF was 57.2% (based on the BFI score) among early-stage non-small cell lung cancer survivors. Based on the findings of previous studies, our findings suggested that EC patients who did not undergo esophagectomy, still may be more vulnerable to CRF than patients with other cancer types. Therefore, more fatigue symptom management could be provided for EC patients.

The present study extended the existing evidence on cancer-related physical symptoms by quantitatively investigating the association between cancer-related physical symptoms and CRF among EC patients. According to our findings, constipation, diarrhoea, and difficulty swallowing were positively related to CRF. Difficulty in swallowing was one of the most common physical symptoms among the EC patients [32]. In the early phase of EC, most patients experience progressive difficulty swallowing, which may result in rapid weight loss [33]. Constipation and diarrhoea, as digestive symptoms are common symptoms caused by medication or chemoradiotherapy [32]. These digestive symptoms may lead to a decrease in nutrient intake, which is linked to weight loss. Increasing studies found that CRF was associated with an impaired nutritional status [34, 35]. Franz et al. [36] also reported that severe weight loss exhibited greater fatigue levels and severe weight loss was an independent predictor of moderate and severe fatigue among old patients. In other words, constipation, diarrhoea, and difficulty swallowing significantly increased the level of CRF due to weight loss or lack of energy or nutrients. Furthermore, these physical symptoms negatively affect quality of life among EC patients [32]. Therefore, to alleviate CRF in EC patients, it is necessary to implement symptom intervention to minimize or eliminate eating and digestive problems.

The present study revealed that depressive symptoms contributed to CRF among EC patients, which is consistent with the findings of other studies [14, 31]. Depressive symptoms are among the most common mental problems in cancer patients. The present study reported that the prevalence of depressive symptoms was 32.0%. Our results were higher than the findings based on the PHQ-9 scores obtained by Zhu et al. (depressive symptoms: 4.16% among Chinese EC patients) [28, 37], which may explain why the prevalence of fatigue in our study was relatively high. Depressive symptoms may elicit negative cognitions (e.g., catastrophizing) that can result in behavioral habituation (e.g., avoiding physical activity), which makes it more challenging to break the cycle of negativity and inactivity [38]. Our findings also revealed that moderate physical activity could relieve CRF in EC patients, possibly because PE can increase muscle strength and physical fitness to counteract physical deconditioning, which directly affects CRF [39]. In turn, PE is valuable for improving psychological outcomes among cancer patients [22]. Additionally, a previous study revealed that negative emotional symptoms were present in 61.6% of fatigued patients, suggesting that, at least in part, fatigue may share the same pathogenic mechanism of depression and psychological disorders [40].

### Implications

Given the above findings, several practical implications should be highlighted. First, the relatively high prevalence of CRF suggests that it is urgent to routinely screen all EC patients for clinically significant CRF. Second, our study suggested that addressing the problem of physical symptoms is vital for EC patients with moderate-severe CRF in clinical nursing. Notably, such symptom management should prioritize managing constipation, diarrhoea, and difficulty swallowing symptoms among EC patients because these symptoms were significantly associated with CRF in the present study. Finally, if moderate-severe fatigue is present, depressive symptoms should also be assessed among EC patients. When a negative mood is present, psychological treatment should be provided, including psychotherapy (e.g., cognitive-behavioral therapy) [41] or the administration of medication (e.g., antidepressants with dual anxiolytic effects) [42].

### Limitations

This study has several limitations. First, this was a cross-sectional study, and causal conclusions of the relationship between these influencing factors and CRF in EC patients could not be drawn. Therefore, further research should use a longitudinal design to confirm these associations. Second, convenience sampling was used in the present study. In addition, the significant associations between variables may be affected by the relatively small sample size, which limits the representativeness of the study. Therefore, the results and conclusions of this study should be interpreted carefully. Third, our study failed to compare the prevalence of CRF between EC patients who have undergone surgery and those who have not due to small sample size.

Esophagectomy is a surgery associated with high postoperative complication rates and may increase the risk of CRF postoperatively. Therefore, further research should be conducted to compare the level of CRF among different treatment methods for EC patients.

## Conclusions

This study revealed a high prevalence of moderate-severe CRF among patients with EC without esophagectomy. Physical and psychological symptoms were significant factors for CRF. Specifically, constipation, diarrhoea, and difficulty swallowing contributed to CRF, and depressive symptoms increased the level of CRF among EC patients without esophagectomy. Therefore, our findings emphasize the importance of interventions for physical and psychological symptom management to alleviate CRF in EC patients.

## Data Availability

The dataset in this study is available from the corresponding author on reasonable request.

## Abbreviations

BFI: Brief Fatigue Inventory
CRF: Cancer-related Fatigue
EC: Esophageal Cancer
GAD-7: Generalized Anxiety Disorder-7
MDASI-GI: MD Anderson Symptom Inventory Gastrointestinal Cancer Module
SD: Standard Deviation
